# Antimicrobial Activity of Positively Charged Oligopeptides with Theoretical High α-Helix Content against *Cutibacterium acnes*

**DOI:** 10.3390/ijms25137445

**Published:** 2024-07-06

**Authors:** Miyako Yoshida, Saki Hayashi, Tamami Haraguchi, Momoka Ito, Yoshiro Hatanaka, Miki Yoshii, Hiroaki Tatsuoka, Shigemitsu Tanaka, Toshihiro Nagao

**Affiliations:** 1 Department of Clinical Pharmaceutics, Faculty of Pharmaceutical Sciences, Mukogawa Women’s University, 11-68 Koshien 9-Bancho, Nishinomiya City 663-8179, Hyogo, Japan; hayashi_saki_x@mukogawa-u.ac.jp (S.H.); tsuchiko_tamami_x@mukogawa-u.ac.jp (T.H.); 1921924@mwu.jp (M.I.); 2 Osaka Research Institute of Industrial Science and Technology, 1-6-50 Morinomiya, Joto-ku, Osaka City 536-8553, Osaka, Japan; hatanaka@orist.jp (Y.H.); yoshii@orist.jp (M.Y.); tatsuoka.hiroaki@orist.jp (H.T.); s-tanaka@orist.jp (S.T.); nagao@orist.jp (T.N.)

**Keywords:** antimicrobial oligopeptide, *Cutibacterium acnes*, minimum inhibitory concentration, scanning electron microscopy, transmission electron microscopy

## Abstract

*Cutibacterium acnes* is abundant and commonly exists as a superficial bacteria on human skin. Recently, the resistance of *C. acnes* to antimicrobial agents has become a serious concern, necessitating the development of alternative pharmaceutical products with antimicrobial activity against *C. acnes*. To address this need, we evaluated the antimicrobial activity of CKR-13—a mutant oligopeptide of FK-13 with increased net charge and theoretical α-helical content—against *C. acnes* in modified Gifu Anaerobic Medium broth by determining the minimum inhibitory concentration (MIC). CKR-13 exerted greater antimicrobial activity against *C. acnes* than FK-13 in the broth at pH 7.0. The antimicrobial activity of CKR-13 with RXM against *C. albicans* was pH-dependent. The ionization of CKR-13 and pH-dependent growth delay of *C. albicans* was suggested to be associated with the increase in CKR-13 antimicrobial activity.

## 1. Introduction

Peptide drugs are currently used primarily to treat metabolic diseases and cancer. However, the clinical application of peptide drugs to treat infectious diseases and inflammation is increasing [1,2]. Antimicrobial peptides (AMPs) show potencies similar to novel antimicrobial drugs, functioning as host-defence peptides [3]. AMPs accelerate biological activity by interacting with the plasma membrane, disrupting it and causing cell lysis, or by being taken up by the target cell; the type of activity depends on their amino acid composition [4]. AMPs commonly have positive net charge ranging from +3 to +9, have an amphipathic and cationic structure, and comprise 12–50 amino acid residues [5,6,7,8]. They exhibit broad-spectrum activity, making them useful for targeting several pathogenic microorganisms.

In particular, derivative AMPs are effective in enhancing antimicrobial activity. A designed hybrid peptide with a combination of α-helix and positive charges has been reported to have more potent antibacterial activity compared with each fragment alone [9]. For hybrid peptides, the α-helical content and number of cations in AMPs are important factors affecting their antibacterial activities.

LL-37 (amino acid sequence LLGDFFRKSKEKIGKEFKRIVQRIKDFLRNLVPRTES) is one of the human AMPs that belongs to the cathelicidin family [10] with an amphipathic α-helical content. This positively charged peptide comprises 37 amino acids and shows antimicrobial activity [11,12,13]. LL-37 and its substituted fragment, which comprise bactericidal core peptides, are suggested to accelerate antimicrobial activity, and their production is cost-effective [14,15]. We have previously designed the antimicrobial oligopeptide CKR-13 (amino acid sequence CKRIVKRIKKWLR), which is substituted for FK-13 (amino acid sequence FKRIVQRIKDFLR), the active antimicrobial fragment of LL-37. CKR-13 exhibited higher net charge than other peptides (LL-37 and FK-13), higher theoretical α-helical content than LL-37, and the same theoretical α-helical content as FK-13 [16].

*Cutibacterium acnes*, a Gram-positive and anaerobic bacterium, is abundant and commonly exists as a superficial bacteria on human skin [17]. It occurs predominately in sebaceous areas and plays an important role in the pathogenesis of *acne vulgaris* and implant-associated infections. *C. acnes* is known to exist in the meibomian gland, which is the sebaceous gland of the eyelid. Infection of the meibomian gland, chronic blepharitis, or endophthalmitis is caused by *C. acnes* [18,19,20]. Acne mostly affects the skin on the upper body, including the face, with non-inflammatory or inflammatory lesions and seborrhoea, and results in varied degrees of scarring. Acne affects approximately 9.4% of the world’s population, including 85% of teenagers and young people. Despite acne being one of the most prevalent diseases worldwide, the function of *C. acnes* in the human body remains partially unclear [21,22,23,24].

*Cutibacterium acnes* participates in the maintenance of skin homeostasis. Three subspecies, *C. acnes* subsp. *acnes*, *C. acnes* subsp. *defendens*, and *C. acnes* subsp. *elongatum*, associate with these subspecies and have recently been suggested to correlate with acne, prostate cancer, and progressive macular hypomelanosis, respectively. Different phylotypes or clonal complexes may cause infections at prosthetic joints and other areas, and virulence factors such as fimbriae, biofilms, and multidrug-resistant plasmids. Production of porphyrin—a *C. acnes* metabolite, which causes acne vulgaris in the skin—and cytotoxicity contribute to infections. Resistance of acneic strains was shown to macrolides (25.0–73.0% of strains resistant), clindamycin (10.0–59.0% of strains resistant), and tetracyclines (up to 37.0% of strains resistant). Sarecycline, AMPs, and bacteriophages may be novel therapeutic antimicrobial drugs [25].

To address the problem of resistance, we evaluated the antimicrobial activity of CKR-13 against *C. acnes* in modified Gifu Anaerobic Medium broth at pH 7. We evaluated its antimicrobial activity in combination with roxithromycin (RXM, another antimicrobial) and examined the influence of broth pH on its antimicrobial activity.

## 2. Results

### 2.1. Theoretical Parameters of the Structure of Antimicrobial Oligopeptides

First, the structural properties of the peptides were evaluated. Table 1 presents the sequences of amino acids and the predicted secondary structures of FK-13 and CKR-13. Based on these predicted structures, FK-13 and CKR-13 have the same helical content (84.6%). Calculated molecular properties are summarized in Table 1. The theoretical α-helix content was correlated with the location of hydrophobic residue at the N-terminus of peptides, and substitutions of amino acids in CKR-13 caused changes in net charge. CKR-13 exhibited a higher net charge than FK-13.

### 2.2. Minimum Inhibitory Concentration (MIC) of FK-13 and CKR-13 in Modified Gifu Anaerobic Medium Broth at pH 7

We examined the antibacterial activity of FK-13 and CKR-13 in modified Gifu Anaerobic Medium broth at pH 7 using the broth microdilution method (Table 2). At pH 7, FK-13 and CKR-13 had MICs of 200 and 50 µg/mL, respectively, indicating that CKR-13 exhibited more potent antimicrobial activity than FK-13.

### 2.3. SEM

**Figure 1:**
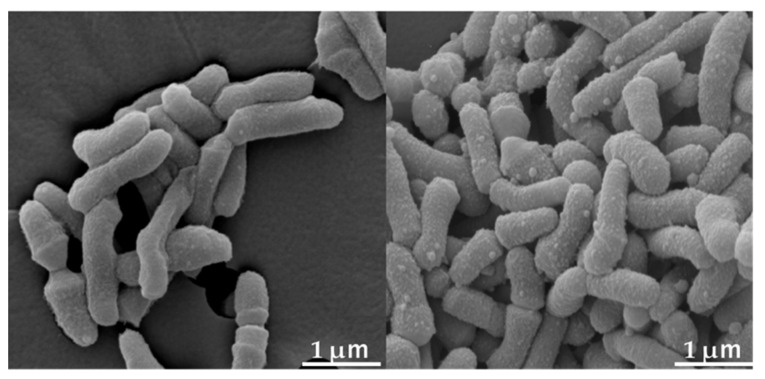
Morphological changes in *Cutibacterium acnes* cells in the absence (**left**) and presence (**right**) of CKR-13 2 h after incubation with CKR-13 (240 µg/mL) as shown by scanning electron microscopy images.

Morphological changes in *C. acnes* were observed using SEM 2 h after incubation with CKR-13 (240 µg/mL). In the presence of CKR-13, the cell surface of *C. acnes* became rough, with the formation of follicles (Figure 1).

### 2.4. TEM

**Figure 2:**
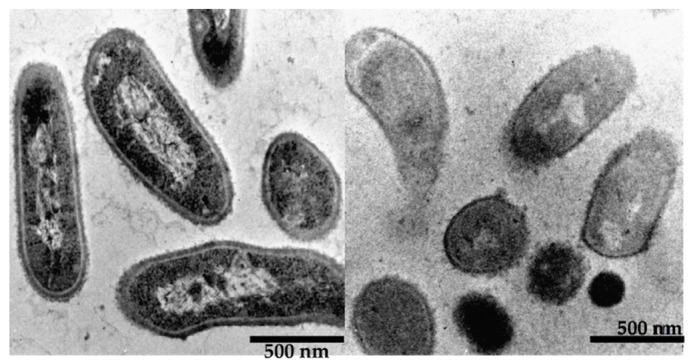
Morphological changes in Cutibacterium acnes cells in the absence (**left**) and presence (**right**) of CKR-13 2 h after incubation with CKR-13 (240 µg/mL) as shown by transmission electron microscopy.

Morphological changes in *C. acnes* were observed using TEM 2 h after incubation with CKR-13 (240 µg/mL). CKR-13-treated cells exhibited deformed surfaces, with the formation of follicles (Figure 2).

### 2.5. MIC of CKR-13 and RXM (Separately and in Combination)

We examined the antibacterial activity of CKR-13 and RXM, separately and in combination, in modified Gifu Anaerobic Medium broth at pH 7 using the broth microdilution method (Table 2). Separately, CKR-13 and RXM exhibited MICs of 50 and 25 ng/mL, respectively; in combination, their MICs were lower, at 12.5 µg/mL and 12.5 ng/mL, respectively. This suggests that the antimicrobial effects of CKR-13 and RXM are synergistic.

### 2.6. Effects of pH on the MIC of CKR-13 with RXM in Modified Gifu Anaerobic Medium Broth

**Figure 3:**
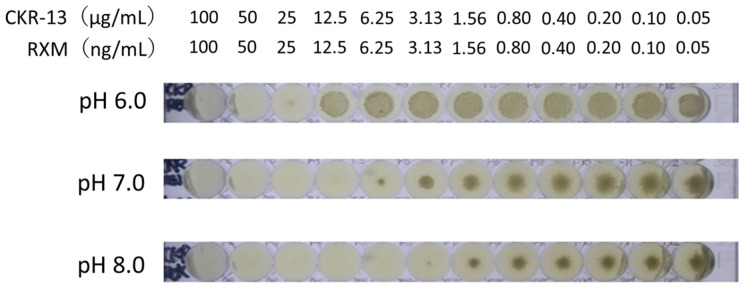
Effects of the pH of modified Gifu Anaerobic Medium broth on the antimicrobial activity of CKR-13 with RXM.

The broth microdilution method revealed that the MICs of CKR-13 in modified Gifu Anaerobic Medium broth at pH 6, 7, and 8 were 200, 50, and 25 µg/mL, respectively. The broth microdilution method revealed that the MICs of RXM in modified Gifu Anaerobic Medium broth at pH 6, 7, and 8 were 100, 25, and 25 ng/mL, respectively. Furthermore, the broth microdilution method revealed that the MICs of CKR-13 with RXM in modified Gifu Anaerobic Medium broth at pH 6, 7, and 8 were 50 µg/mL with 50 ng/mL, 12.5 µg/mL with 12.5 ng/mL, and 6.25 µg/mL with 6.25 ng/mL, respectively (Figure 3). This reveals that the antimicrobial activity of CKR-13 increased as pH increased. A pH-dependent growth delay of *C. acnes* was observed. The growth delay of pH-dependent *C. acnes* was suggested to have an influence on the antibacterial activity of CKR-13 with RXM.

## 3. Discussion

This study examined the antimicrobial activity of CKR-13 with broad-spectrum activity [16]. Structure–activity modelling to determine the structural features responsible for the activity of tethered peptides has revealed that the magnitude of the positive charge and the location of the hydrophobic residues within the molecule affect AMP activity [26]. We designed CKR-13 using the Antimicrobial Peptide Database (https://aps.unmc.edu/; Last accessed: 20 December 2023). NetSurfP-2.0 (https://services.healthtech.dtu.dk/services/NetSurfP-2.0/; last accessed 20 December 2023) was used to evaluate its physical and chemical properties (α-helix content (%) and net charge). It is known that the pKa of the ε-amino group of the lysin residue is 10.5 and the pKa of the guanidino group of the arginine residue is 12.5. From pH 6 to 8, most of the lysine and arginine in the amino acid sequence of AMP were suggested to be ionized. The positive charge is closely associated with the electrostatic binding force between the AMP and the negatively charged bacterial cell membrane [27,28]. In this study, the increase in positive charge in the amino acid sequence in CKR-13 was suggested to correlate with potent antimicrobial activity. The α-helix structure may enable deep insertion of the AMP into the bacterial cell membrane [29,30]. Our findings provide evidence that the helical structure can affect bacterial cells. Remarkably, a cysteine residue at the N-terminus of CKR-13 (amino acid sequence CKRIVKRIKKWLR) showed greater helical structural content (85%) than a cysteine residue that was shifted to the C-terminus from the N-terminus of CKR-13 (amino acid sequence KRIVKRIKKWLRC) (77%), although the value of positive charges did not differ between these two peptides. The location of the cysteine, which is a hydrophobic residue, influences the content of the α-helical structure of CKR-13. Thus, CKR-13 exhibited potent antimicrobial activity. The antimicrobial activity against *C. acnes* was suggested to depend on the structural and physicochemical properties of AMP.

CKR-13 is an FK-13-mutant oligopeptide with a higher net charge and the same theoretical α-helical content as FK-13. At pH 7, CKR-13 exhibited greater antimicrobial activity against *C. acnes* than FK-13. CKR-13 exhibited greater antimicrobial activity in combination with RXM than alone, suggesting that CKR-13 and RXM exhibit synergistic antimicrobial activity.

RXM, a macrolide antibiotic, possesses good antibacterial activity against the anaerobic *C. acnes*. In clinical practice, RXM is frequently used as an antimicrobial agent against *C. acnes*. Furthermore, it exerts senolytic effects, eliminating senescent MRC-5 cells [31]. In mice, RXM reduced airway inflammation in airway injury to induce asthma and in bleomycin-induced acute lung injury [32,33,34]. In patients with non-cystic fibrosis bronchiectasis, low-dose, long-term use of RXM ameliorated remodelling of dilated bronchial walls [35]. RXM inhibited fibroblast activation in vitro and attenuated bleomycin-induced pulmonary fibrosis and the senescent phenotype during pathogenesis in vivo. RXM potentially exerts these effects by downregulating the expression of NADPH oxidase 4, which is implicated in cell senescence and lung fibrosis.

We have previously reported the antimicrobial effects of CRK-13 against *S. aureus*, *E. coli*, and *C. albicans* [16]. Generally, antimicrobial peptides act on the pellicle surface of bacteria. In the current study, we observed morphological changes in *C. acnes* caused by CKR-13 using SEM and TEM. In our prior work, SEM and TEM revealed morphological changes on the bacterial surface of *S. aureus*, *E. coli*, and *C. albicans* in the presence of high-concentration CKR-13 conjugate at 2 h [16]. According to our previous work, SEM and TEM revealed that the surface of *C. acnes* exposed to extremely high concentrations compared to MICs (CKR-13 incubation time: 48 h) of CKR-13 exhibited morphological changes after 2 h of incubation with CKR-13. Therefore, we assume that CKR-13 acts by attacking the bacterial cell surface. The synergistic antibacterial action of CKR-13 and RXM may arise from differences in their mechanisms of action.

CKR-13 was prepared from FK-13 by replacing some of its basic amino acids, such as lysine or arginine, to increase the positive charge. CKR-13 exhibited more potent activity than FK-13 against *C. acnes*. Their theoretical α-helical content was the same. From pH 6 to 8, more lysine and arginine residues in the amino acid sequence of AMP were suggested to be ionized because the pKa of lysine is 10.5 and the pKa of arginine is 12.5. The higher positive charge of CKR-13 compared to FK-13 is potentially the primary factor contributing to its antimicrobial activity against *C. acnes.* LL-37, which contains FK-13 in its structure, also has antimicrobial activity against *C. acnes* [36].

Broth pH was identified as a determining factor for CKR-13 antimicrobial activity. At pH 7, CKR-13 with many ionized basic amino acid residues showed potent antimicrobial activity against *C. acnes* compared to FK-13 with few ionized basic amino acid residues. The ionization of basic amino acid residues such as arginine and lysine in CKR-13 was suggested to occur at pH 6, 7, and 8 because the pH value was smaller than the pKa of the basic group of basic amino acid residues (the pKa of the ε-amino group of the lysine residue is 10.5 and the pKa of the guanidino group of the arginine residue is 12.5).

The antimicrobial activity of CKR-13 exhibited stronger broth pH dependency than that of FK-13. Both FK-13 and CKR-13 exhibited pH-dependent antimicrobial activity; therefore, we suggest that the pH-dependent increase in antimicrobial activity of CKR-13 is not associated with the charge of the peptide. As the growth of *C. acnes* depends on the pH, antibacterial activity might become relatively higher.

In this study, the combination of CKR-13 and RXM decreased the MICs of CKR-13 and RXM. The pH-dependent decrease in the MIC of RXM was not observed at pH 7 and 8. However, pH-dependent decreases in the MICs of CKR-13 and RXM were observed at pH 7, 8, and 9. CKR-13 was suggested to act as a booster for RXM.

Oxarol^®^ Ointment (active ingredient: maxacalcitol), an approved drug in Japan, has a pH of 8–9. Therefore, a preparation of CKR-13 for dermal application could have a pH of 8.

Further research is needed to clarify the pH dependency of antimicrobial activity.

## 4. Materials and Methods

### 4.1. Materials

The LL-37 oligopeptide fragment (17–29), FK-13, was purchased from Funakoshi Co., Ltd. (Tokyo, Japan). CKR-13, a substituted oligopeptide derived from FK-13, was prepared on commission by Toray Research Center (Tokyo, Japan). The microbial strain was purchased from the Biological Resource Center of the National Institute of Technology and Evaluation (NBRC, Tokyo, Japan). The strain used here was *C. acnes* subsp. *acnes* NBRC 107605^T^ (referred to here as *C. acnes*).

### 4.2. Determination of MICs

The antibacterial activity levels of FK-13, CKR-13, RXM, and CKR-13 and RXM combined were characterized by determining their MICs. Compound solutions were prepared using Modified Gifu Anaerobic Medium broth (Shimadzu Diagnostics, Kyoto, Japan). The final concentrations of FK-13 and CKR-13 in solution were 0.10, 0.20, 0.39, 0.78, 1.56, 3.13, 6.25, 12.5, 25, 50, 100, or 200 μg/mL. For RMX alone, they were 0.10, 0.20, 0.39, 0.78, 1.56, 3.13, 6.25, 12.5, 25, 50, 100, and 200 ng/mL. The concentrations of CKR-13 (μg/mL) and RXM (ng/mL) in the CKR-13 and RXM combination were, respectively, 0.05 and 0.05, 0.10 and 0.10, 0.20 and 0.20, 0.40 and 0.40, 0.80 and 0.80, 1.56 and 1.56, 3.13 and 3.13, 6.25 and 6.25, 12.5 and 12.5, 25 and 25, 50 and 50, and 100 and 100. The *C. acnes* strain in the exponential growth phase was diluted to 4 × 10^4^ CFU/mL in Modified Gifu Anaerobic Medium broth at pH 7 (for FK-13, CKR-13, RXM, and the CKR-13 and RXM combination) and at pH 6 or 8 for CKR-13. After 150 µL of the culture was dispersed into each well of a 96-well microtiter plate, the plates were incubated for 48 h at 37 °C under anaerobic conditions. MIC was determined as the minimum inhibitory concentration of the compound. A clear well indicated that bacterial growth had not occurred.

### 4.3. SEM

Morphological observation of C. acnes using SEM was conducted as described previously [37], with minor modifications. *C. acnes* cells were cultured to the stationary phase (4.0 × 10^9^ CFU/mL) in Mueller–Hinton broth. The cell suspension (4 × 10^9^ CFU/mL) was incubated with CKR-13 (240 µg/mL) at 27 °C for 2 h. Next, *C. acnes* cells were fixed at 4 °C with 2.5% glutaraldehyde overnight. After washing twice with PBS, *C. acnes* cells were post-fixed with 1% OsO_4_ for 2 h. Cells were dehydrated via a graded ethanol solution series, coated with gold, and observed using SEM (JSM-7800, Jeol Ltd., Tokyo, Japan).

### 4.4. TEM

Morphological observation of *C. acnes* using TEM was conducted as described previously [36], with minor modification. *C. acnes* cells were cultured to the stationary phase (4.0 × 10^9^ CFU/mL) in Mueller–Hinton broth and incubated with CKR-13 (240 µg/mL) at 27 °C for 2 h. After incubation, glutaraldehyde was added in the form of 2.5% glutaraldehyde solution. After centrifugation of the cells, they were embedded in 3% low-melting-point agar and post-fixed for 1 h in 4% buffered OsO_4_. After washing three times with ethanol, the cells were embedded in epoxy resin (Quetol 651 Mix). Ultrathin (100 nm) sections prepared using the ultramicrotome were mounted on the copper mesh. Finally, specimens counterstained with 2% (*w*/*v*) uranyl acetate solution (20 min) and Reynolds lead solution (5 min) were analysed using TEM (JEM2100, JEOL Ltd., Tokyo, Japan).

## 5. Conclusions

The antimicrobial oligopeptide CKR-13 designed in this study was substituted from FK-13, a partial fragment containing amino acid numbers 17–29 of an amino acid sequence of human antimicrobial peptideLL-37. CKR-13 exhibited a higher net charge than FK-13 but the same theoretical α-helical content. CKR-13 exerted greater antimicrobial activity against *C. acnes* than FK-13 in modified Gifu Anaerobic Medium broth at pH 7 (the standard pH for this broth). From the observations made using SEM and TEM, CKR-13 was suggested to attack the surface of *C. acnes* cells, leading to follicle formation on their surfaces. At pH 7, CKR-13 exerted greater antimicrobial activity in combination with RXM than alone, suggesting that CKR-13 and RXM exert synergistic antimicrobial activity. The antimicrobial activity of CKR-13 with RXM against *C. albicans* was pH-dependent. The ionization of basic amino acids and pH-dependent growth delay of *C. albicans* were suggested to be associated with the increase in CKR-13 antimicrobial activity. CKR-13 may be a useful antimicrobial peptide due to its high antimicrobial activity against *C. acnes*.

## Figures and Tables

**Table 1:** Parameters of the theoretical structures of FK-13 and CKR-13.

Antimicrobial Oligopeptide	Amino Acid Sequence	α-Helix Content (%)	Net Charge
FK-13	FKRIVQRIKDFLR	84.6	+4
CKR-13	CKRIVKRIKKWLR	84.6	+7

**Table 2:** Minimum inhibitory concentrations (MICs) of FK-13, CKR-13, RXM individually, and CKR-13 and RXM combined against *Cutibacterium acnes* in modified Gifu Anaerobic Medium broth at pH 7.

	MIC			
Microorganism	FK-13	CKR-13	RXM	CKR-13 + RXM
*C. acnes* (NRBC 107605^T^)	200 µg/mL	50 µg/mL	25 ng/mL	CKR-13: 12.5 µg/mL RXM: 12.5 ng/mL

## Data Availability

Data are contained within the article.
